# Structural Features of a Conformation-dependent Antigen Epitope on ORFV-B2L Recognized by the 2E4 mAb

**DOI:** 10.1038/s41598-019-52446-5

**Published:** 2019-11-06

**Authors:** Yongzhong Yu, Wenbo Zhao, Qiang Tan, Xue Zhang, Mengyao Wang, Xuyang Duan, Yuanyuan Liu, Zhijun Wu, Jinzhu Ma, Baifen Song, Rui Zhao, Kui Zhao, Zhengxing Lian, Yudong Cui

**Affiliations:** 1 Virology Laboratory, College of Biological Science and Technology, Heilongjiang Bayi Agricultural University, 5 Xinfeng road, Daqing, 163319 China; 2 Pharmacology laboratory, Heilongjiang Bayi Agricultural University, 5 Xinfeng road, Daqing, 163319 China; 3 College of Animal Science and Veterinary Medicine, Jilin University, 5333 Xi’an Road, Changchun, 130062 China; 4 Beijing Key Laboratory for Animal Genetic Improvement, College of Animal Science and Technology, China Agricultural University, Beijing, 100193 China

**Keywords:** Antibodies, Antibodies, Antivirals, Antivirals

## Abstract

Previously, we successfully prepared a monoclonal antibody (mAb) named 2E4, that directly recognizes the major envelope protein B2L of the orf virus (ORFV), but there is little information about its epitope. Here, we meticulously mapped the 2E4 epitope through combinatorial programs and identified the functional binding domain and a key amino acid residue. Briefly, the simulated epitope peptide closely resembles ^84^VDVQSKDKDADELR^97^ located at the N-terminus of B2L, strongly suggesting that the epitope is conformationally or spatially structure-dependent. Subsequently, we combined these findings with the results from the antigenicity prediction of B2L to design three truncated fragments of B2L (F1, F2 and F3) selected using 2E4, and only the F1 fragment was found to be eligible for the advanced stage. Alanine-scanning mutagenesis suggested that the D^94^ residue is structurally crucial for the 2E4 epitope. The other participating residues, including K^61^, E^62^, and D^92^, together with D^94^ were responsible for enabling 2E4 binding and served as factors that synergistically enabled binding to the whole 2E4 epitope. In this paper, we describe, for the first time, the architecture of an ORFV conformational epitope, and it is also expected that mAb 2E4 and its epitope can be used for applications relating to orf control.

## Introduction

Orf is a zoonosis that is usually transmitted from affected sheep or goats through direct contact or contaminated fomites^1^. ORFV, a species from the family *Poxviridae* and genus *Parapoxvirus* (PPV), is responsible for contagious ecthyma, mainly leading to a partial epitheliotropic effect via broken or scarified skin and gives rise to pustular lesions^2,3^. The aetiology of this condition is shown to be the presence of *poxvirus*, which can be identified in clinical samples through the use of electron microscopy, serological methods, polymerase chain reaction (PCR) (a powerful tool, especially for discriminating diseases from the similar clinical symptoms, e.g., orf and foot and mouth disease), and genetic analysis of unique genes, such as *B2L*, which is encoded by ORFV^4,5^. The *B2L* gene contains an open reading frame of 1137 bp that encodes a putative polypeptide of 378 amino acids that is known to be the primary immunogenic envelope protein p42K, the homologue of p37K from the vaccinia virus^6^. Additionally, orf virus, pseudocowpox virus (PCPV) and bovine popular stomatitis virus (BPSV) are all PPVs with high sequence fidelity in the *B2L* gene, which suggests that the *B2L* gene may be a molecular marker in PPV DNA (Fig. s1). Observing this point, we designed a semi-nested PCR^7^ and a loop-mediated isothermal amplification (LAMP) assay^8^ to detect viral DNA for orf lab-diagnosis. Based on the *B2L* gene, a real-time quantitative PCR assay has been developed for ORFV DNA quantification in infected cells or organic cultures^9^. However, another helpful tool for orf detection is using serological methods to assess antigen-antibody interactions. Binding is available to address antigenic sites, to track mutants^10^ for passive immunotherapy^11^ (such as neutralization) and to provide a protection strategy in patients, such as using an epitope vaccine^12–15^.

The method used to map antigenic determinants is important for investigating the mechanism by which antigens and antibodies externally assemble in a biological process. Significantly, epitope mapping is helpful in vaccine design. A B-cell epitope or paratope often acts as basic data and is defined by its complementary and adaptive potential as well as its activity^16^. In 1993, a series of synthetic peptides derived from proteins encoded by open reading frames 2 and 3 (ORF2 and ORF3) of the hepatitis E virus were used in an enzyme immunoassay to determine the localization of an epitope^17^. Soon after that, phage-displayed random peptide libraries were shown to be a promising technique for the investigation of protein-protein interaction. By screening peptide mimics from a random biological protein molecule library, researchers have broken through many barriers in the methodology of epitope mapping. This process facilitates the discovery of antibody-binding fractions or epitopes^18–20^. In 1994, Wang *et al*.^21^ used a recombinant phage expression library to screen for an mAb-defined linear epitope. Until 2011, a conformation-dependent epitope was precisely mapped by a potent neutralizing mAb 3E11, which could neutralize the foot-and-mouth disease virus (FMDV) Asia1/YS/CHA/05 *in vitro*^22^. Other applications of the immunodominant epitopes have been reported in serodiagnosis and vaccine development^23^.

In our previous study, we prepared three mAbs against ORFV B2L, and only the mAb 2E4 was found to exhibit certain neutralization activity to ORFV infection *in vitro*. In addition, 2E4 was able to specifically recognize ORFV in an indirect enzyme-linked immunosorbent assay (ELISA) and an indirect immunofluorescent assay (IFA) but did not recognize epitopes according to western blotting (in which it did not recognize denatured protein). Given that there may be further applications of 2E4, this study aimed to better characterize the epitope of interest to which the mAb for 2E4 (mAb 2E4) binds and employed a set of integrated approaches to address its unclear topographic profile. Detailed information related to the 2E4 epitope will assist us by providing with a better ability to combat this viral disease in the future.

## Results

### Mock epitope recognized by mAb 2E4 via biopanning

**Figure 1:**
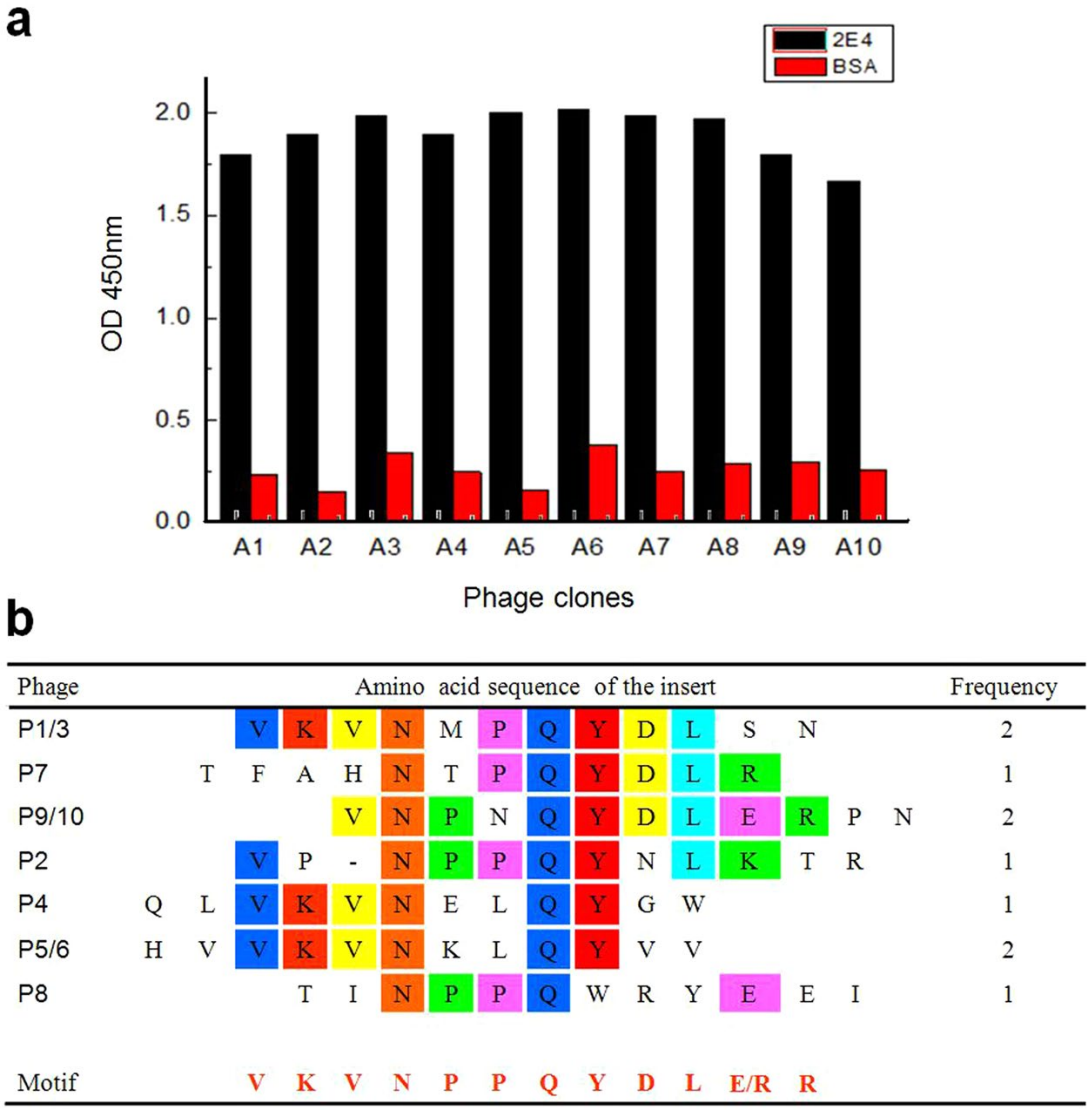
Phage ELISA and sequence comparison of random peptide inserts presented in the positive phage clones. (**a**) A total of 10 phage samples (P1 to P10) had various positive values as indicated by ELISA; however, the BSA controls displayed lower specificity to mAb 2E4. (**b**) Sequence comparisons of positive phage clones. Conserved amino acid motifs are colored in various kinds of square blocks, the consensus motif VKVNPPQYDLE/RR is shown at the bottom using light red letters, and E/R is indeterminate. Frequency represents the sequence specificity that occasionally repeats within all of the 10 probable samples.

Biopanning of a phage display 12-mer random peptide library was initially performed to determine the mimotope recognized by mAb 2E4 by using the affinity-purified mAb. After four rounds using the biopanning program, individual phage clones were isolated, and the reactivity of these phage clones was assessed^24^ by phage ELISA (Fig. 1a). Ten positive clones were submitted to Sangon Biotech (Shanghai) for sequencing, and final sequences of seven clusters of the phage inserts were collected. Among these, P3, P6 and P10 were repeated samples. Sequence alignment of random peptides resulted in a derived sequence motif: VKVNPPQYDLE/RR (Fig. 1b), where this motif was highly inclined to represent a veritable 2E4 epitope.

### Antigenicity prediction and homology modelling suggested that the 2E4 epitope is located on the B2L-F1 segment

**Figure 2:**
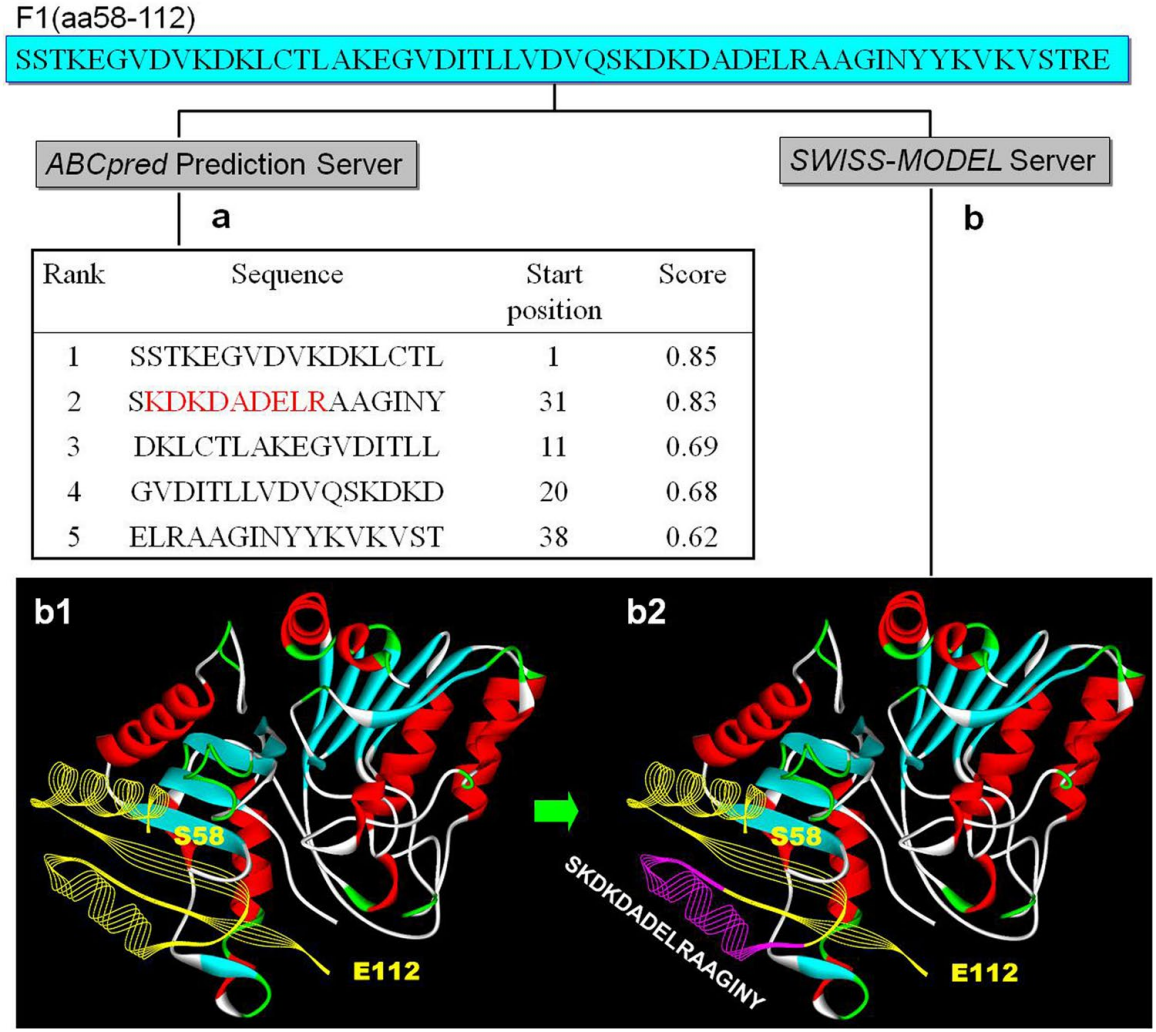
B-cell epitope prediction and homology modelling. (**a**) B2L-F1 is composed of 55 amino acid residues ranging from S58 to E112, and the sequence was submitted to the *ABCpred* Prediction Server for predicting probable B-cell epitopes of mAb 2E4.The server provided 5 referenced options, and their score values are ranked from 0.85 (the highest) to 0.62 (the lowest) via strict selection using an artificial neural network. Among them, No.2 SKDKDADELRAAGINY closely resembles our expectations. The red letters represent a highly putative epitope that includes the candidate residues. (**b**) Homology modelling and 2D structure of B2L via the *SWISS MODEL* server. F1 contains 2α-helical structures in this model. (**b-1**) includes S58~E112, which is displayed ahead of B2L, marked in yellow, and (**b-2**) includes the peptide No. 2 derived from (**a**) at the most prominent location in the F1 peptide fragment marked in pink, which may be responsible for binding to 2E4.

In our previous study, the full length of B2L was artificially partitioned into three truncated peptide fragments: F1 (at aa58-112), F2 (at aa205-235) and F3 (aa at 338-375), which were used for determining the location of the 2E4 epitope. This strategy was based on the results from the antigenicity prediction of B2L obtained using the *DNAStar Protean* program (data not shown here). When the amino acid sequence of F1 (at aa58-112) was submitted to the *ABCpred* Prediction Server, the program gave us some predicted candidate epitope information with various score values. Among them, we found that the first ^58^SSTKEGVDVKDKLCTL^73^ and the second ^88^SKDKDADELRAAGINY^103^ represented the residues located near the N-terminal domain of the F1 segment (Fig. 2a, No.1–2). Subsequently, these two oligopeptides were displayed on the forepart of the 2D structure using the *SWISS MODEL* server (Fig. 2b1–2). ^58^SSTKEGVDVKDKLCTL^73^ and ^88^SKDKDADELRAAGINY^103^ happen to be adjacent to each other topographically, and have some similarity to the VKVNPPQYDLE/RR mimotope. Therefore, we assumed that the authentic epitope recognized by mAb 2E4 was in the B2L-F1 segment.

### B2L-F1 is responsible for 2E4 binding at a conformation-dependent epitope

**Figure 3:**
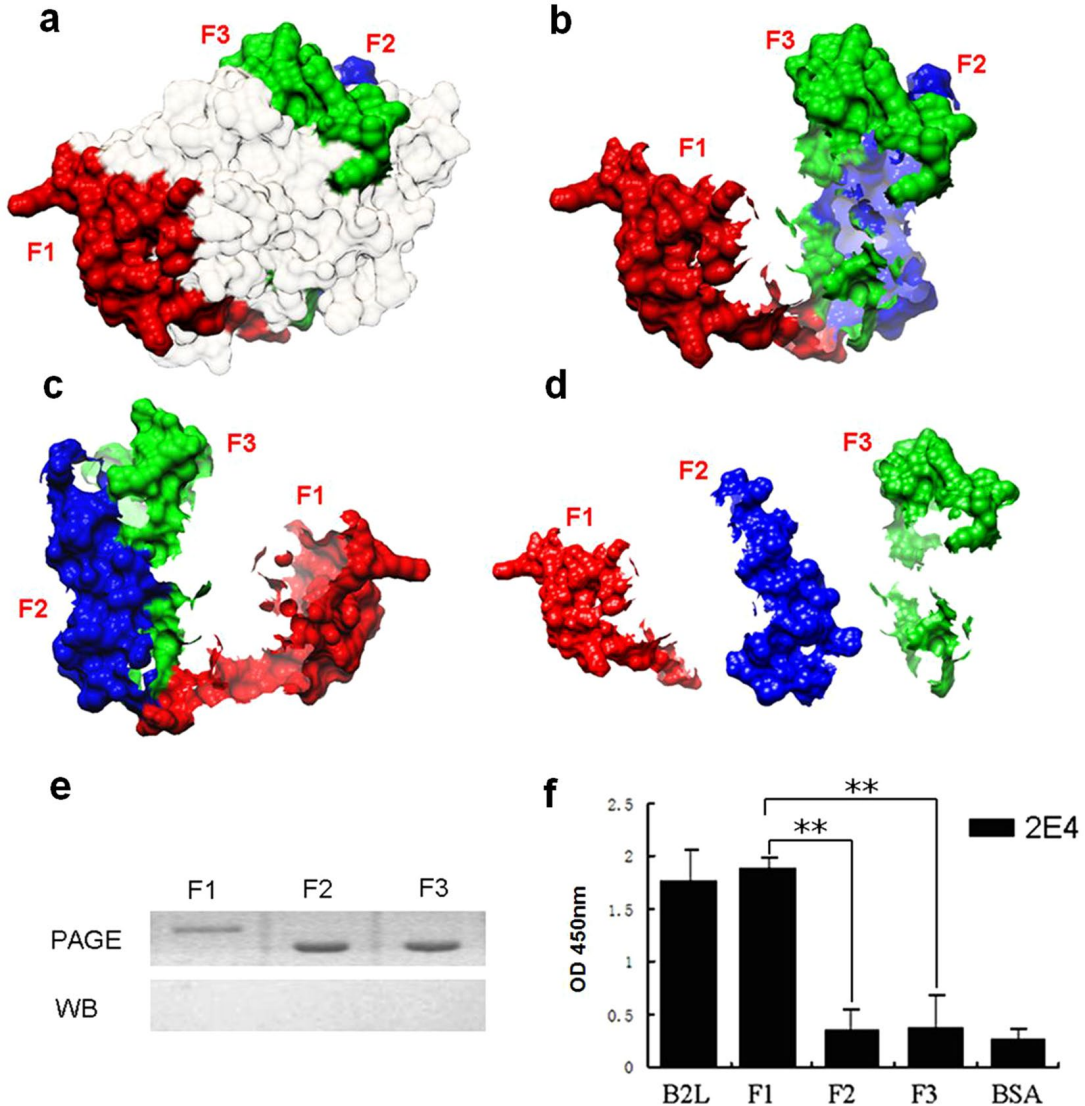
Address determination of the 2E4 epitope. (**a**) The 3D structure of the entire B2L protein was generated via the SWISS-MODEL server and contains three selected regions: F1 (aa58-112 indicated in red), F2 (aa 205-23 indicated in blue) and F3 (aa338- 375 indicated in green). (**b**) Three peptides of interest in B2L. (**c**) The back view of (**b**). (**d**) The separate states of F1, F2 and F3. (**e**) SDS-PAGE and western blot of the three fragments. The GST-fused peptides F1, F2 and F3 were separated by 12% SDS-PAGE. Western blot analysis with 2E4 corresponded with the fusion proteins shown by PAGE, but none of them showed an obvious band on the nitrocellulose membrane. (**f**) F1 was found to specifically recognize mAb 2E4 by ELISA. OD450 nm values less than 0.5 were considered non-specific binding to 2E4 except the full-length B2L antigen and F1 in this ELISA assay. (**p < 0.01, represents a statistically highly significant difference; Mean ± SD, n = 3).

*E.coli* BL21 (DE3) cells containing F1, F2 or F3 recombinant plasmids were induced, and expressed recombinant proteins were purified from a biomass of 0.3 L culture using Ni-NTA agarose (Qiagen, Germany). Approximately equal amounts of *E.coli* cell lysates of expressed GST-fusion proteins were prepared to be electrophoretically separated using a 12% polyacrylamide gel for SDS-PAGE. Nevertheless, no band was found on the nitrocellulose membrane by western blot analysis (Fig. 3e). Fortunately, in ELISA, B2L (Fig. 3a) and F1 (Fig. 3b–d) were recognized by 2E4, whereas both F2 and F3 showed lower activity to 2E4 (Fig. 3f). These results indicated that the 2E4 epitope was located in the F1 region, with a novel discontinuous conformational antigenic determinant, corresponding to our expectations.

### The critical residue of mAb 2E4 epitope was D^94^

**Figure 4:**
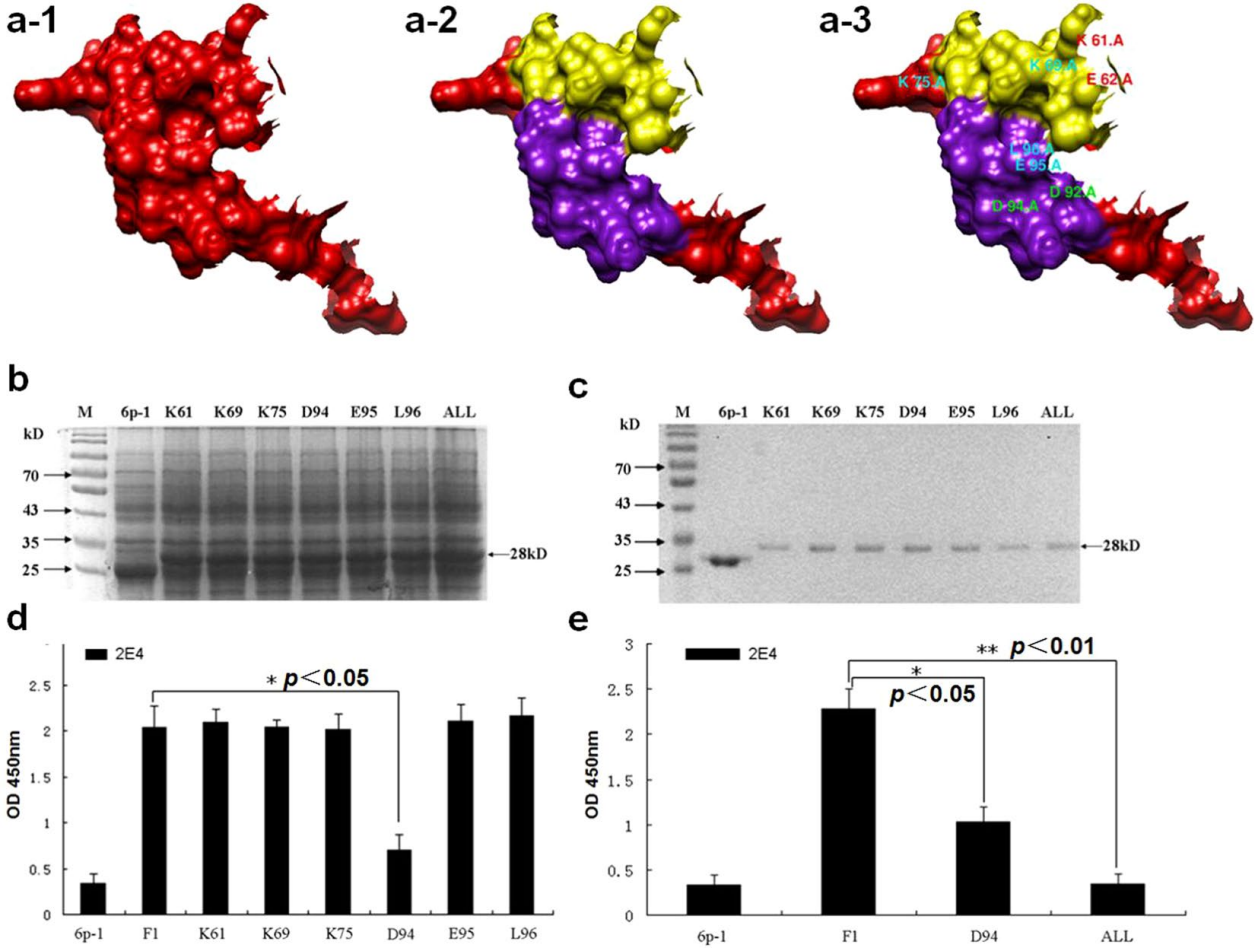
Contributions of a critical residue to the mAb 2E4 epitope. **(a-1)** 3D model of the F1 fragment (in red). **(a-2)** Display of 2α-helical structures on the F1 fragment (in purple and yellow). **(a-3)** All amino acid residues of interest in the 2α-helical structures. **(b)** Approximately equal amounts of *E. coli* cell lysates with the expressed GST-fusion proteins were electrophoresed on a 12% SDS-PAGE gel and stained with Coomassie blue. 6p-1 represents the pGEX-6p-1 vector; K^61^, K^69^, K^75^, D^94^, E^95^, and L^96^ represent individual mutants in which the indicated amino acids were substituted with alanines; “All” represents the mutant in which K^61^, E^62^, D^92^ and D^94^ were simultaneously substituted. **(c)** Expression products are exhibited at approximately 28 kD. The purified expression products were in accord with the situation in (**b**) and used to investigate the reactivity of the variants if the interesting residues were deficient. **(d)** Individual substitutive mutants with reactivity to mAb 2E4 were evaluated by ELISA. The pGEX-6p-1 vector was used as a negative control. Only D^94^ showed remarkably attenuated reactivity to 2E4, contrasting with F1 and other mutant objects. **(e)** “ALL” fully abolished the reactivity of the epitope to 2E4, in contrast with the D^94^ mutant. F1 is a positive control in this test (*p < 0.05, represents a statistically significant difference; **p < 0.01, represents a statistically highly significant difference; Mean ± SD, n = 3).

We were particularly interested in determining whether amino acid changes in the 2E4 epitope could hinder antibody binding. A panel of GST-fusion proteins expressing variants derived from the F1 (aa at 58-112) was generated according to the results of homology modelling of B2L by *SWISS-MODEL* (Fig. 3a) to determine if a particular residue was critical for mAb 2E4 to recognize the epitope, and each individual residue of interest, including K^61^, K^69^, K^75^, D^94^, L^95^ or E^96^, was substituted with an alanine (Fig. 4a1–3; Table 1). PAGE and anti-GST detection suggested all target proteins were successfully expressed (Fig. 4b,c). Among these mutants, GST-D^94^ was found by ELISA to show obviously decreased reactivity to mAb 2E4 (Fig. 4d), suggesting that D^94^ is important for 2E4 binding. The reaction activity of the D^94^ mutant decreased by approximately 70%, while the other mutants showed no impact on binding action compared to the D^94^ mutant, where all of the other mutants corresponded to the F1 original.

**Table 1:** Primers used to generate specific mutations in the B2L-F1 gene of the ORFV OV/HLJ04 strain for key amino acid investigation.

Mutants	The Synthesized oligonucleotides
K^61^/A-F	5-C*GGATCC*AGCTCCACC**GCC**GAGGG-3
K^61^/A-R	5-C*CTCGAG*TTCCCGCGTGGACACCT-3
K^69^/A-F	5-C*GGATCC*AGCTCCACCAAGGAGGGCGTCGACGTCAAAGAC**GCC**CTCT-3
K^69^/A-R	5-C*CTCGAG*TTCCCGCGTGGACACCTT-3
K^75^/A-F	5-C*GGATCC*AGCTCCACCAAGGAGGGCGTCGACGTCAAAGACAAGCTCTGCACGCTC**GCC**GCCGAG-3
K^75^/A-R	5-C*CTCGAG*TTCCCGCGTGGACACCTT-3
D^94^/A-F	5-C*GGATCC*AGCTCCACCAAGGAGGG-3
D^94^/A-R	5-C*CTCGAG*TTCCCGCGTGGACACCTTGACTTTGTAGTAGTTGATGCCCGCCGCGCGCAGCTC**GGC**CGC
E^95^/A-F	5-C*GGATCC*AGCTCCACCAAGGAGGG-3
E^95^/A-R	5-C*CTCGAG*TTCCCGCGTGGACACCTTGACTTTGTAGTAGTTGATGCCCGCCGCGCGCAG**GGC**GTCCGC-3
L^96^/A-F	5-C*GGATCC*AGCTCCACCAAGGAGGG-3
L^96^/A-R	5-C*CTCGAG*TTCCCGCGTGGACACCTTGACTTTGTAGTAGTTGATGCCCGCCGCGCG**GGC**CTCGTCCGC-3

Note: *GGATCC*: *BamH*I restriction site; *CTCGAG*: *Xho*I restriction site; **GCC** or **GGC**: triplet codon of alanine.

### K^61^, E^62^ and D^92^ are natural auxiliary components of the whole 2E4 epitope

**Figure 5:**
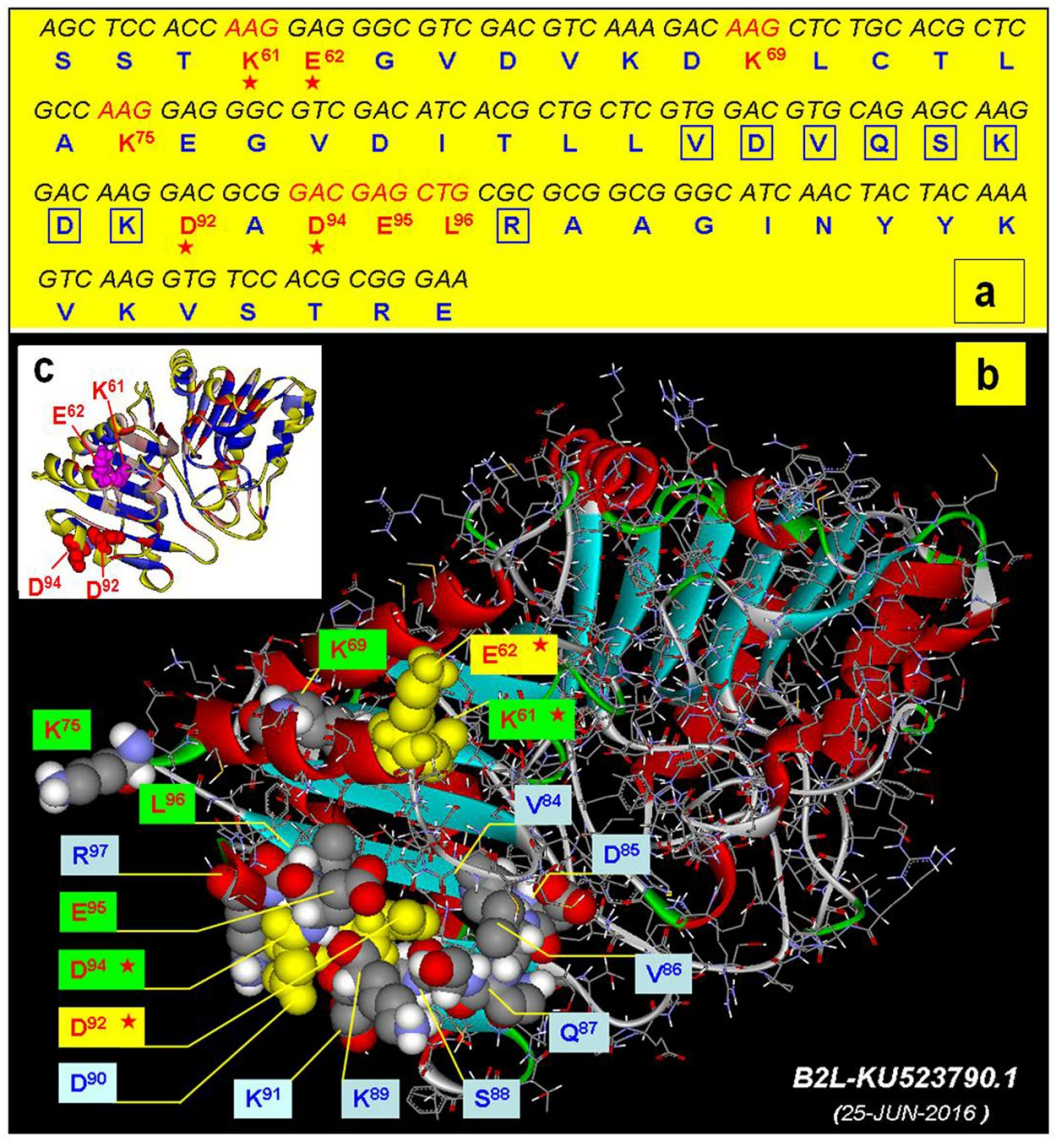
Correlative residues of the mAb 2E4 epitope in the F1 fragment. **(a)** The F1 fragment with nucleotides and their corresponding amino acid sequences. Among these, the most interesting residues are highlighted with red letters and remarkable contributors are marked with a small red star below them. Other doubtful residues surrounding the epitope are boxed with rectangular wire frames. **(b)** The 2D model of the B2L protein with additional atomic bonds (shown in wire). Correlative residues of the mAb 2E4 epitope in the F1 fragment are displayed on the head of the model (shown as a sphere). All residues are divided into three groups (6 residues in green, 2 in yellow and 9 in grey). The highly probable contributor to this epitope is marked with an asterisk. **(c)** The relative position of the major members of the 2E4 epitope. D^92^ and D^94^ are displayed as red spheres, K^61^and E^62^ are displayed as magenta spheres. The α-helix structures are indicated in yellow.

Additionally, based on the B2L crystal structure (homology modelling) and amino acid position of D^94^, we selected K^61^, E^62^ and D^92^ as doubtful contributors to the 2E4 epitope. These three residues, together with D^94^, were simultaneously substituted with alanines, and the mutant K^61^/A-E^62^/A-D^92^/A-D^94^A of F1 was named “ALL” (Fig. 4b). The result showed “ALL” most lacked reactivity compared with the D^94^ mutant alone (Fig. 4d), indicating that the K^61^, E^62^ and the D^92^ residues were natural auxiliary components for mAb 2E4 binding. Summarily, these candidates were evaluated for their ability to affect 2E4 binding directly by alanine substitution. In the N-terminus region of F1, at least 10 exposed amino acids of the F1 surface adjacent to the 2E4 epitope were generated to evaluate their contribution to the 2E4 reactivity of the mutant F1. In contrast, the expressed GST fusion proteins of the mutant F1 peptides, including K^61^, K^69^, K^75^, E^95^ and L^96^, had less impact on binding activity to 2E4 (Fig. 4e). These results indicated that the residue D^94^ was a critical residue of the 2E4 epitope, and taken together, the 2E4 epitope was mapped into an exquisite region containing several original functional residues (Fig. 5a–c).

## Discussion

Epitope information is essential for understanding antigen–antibody interactions^25^. In the present study, we have clarified an accurate conformational epitope for mAb 2E4 binding via several rounds of biopanning^26^. Meanwhile, an epitope MODEL was formulated by online servers and by using a visualization toolkit and was used to help identify the probability distribution of the 2E4 epitope.

Briefly, the biopanning of a random phage display peptide library led to a novel mimotope VKVNPPQYDLE/RR motif (Fig. 1) that was recognized by the mAb 2E4 in phage ELISA. Subsequently, a series of experiments of epitope mapping were designed and achieved. Firstly, segmental expression of B2L was completed according to a B-cell epitope prediction generated beforehand, and every part was singly selected by 2E4. The reactivity of three truncate peptides (the F1, F2 and F3 fragments) were evaluated by western blot and ELISA, followed by alanine scanning mutagenesis combined with online prediction and homology modelling. Finally, mAb 2E4 was experimentally demonstrated to recognize a discontinuous conformational antigenic determinant (binding site) at the N-terminus of the B2L protein of ORFV (Fig. 2). Moreover, D^94^ was identified as a critical residue of this epitope, and could be interpreted as a major factor influencing immunity recognition. Additionally, the K^61^, E^62^, and D^92^ residues within the F1 fragment are required for constructing the epitope of the mAb 2E4, where these residues with D^94^ could likely form the binding region of 2E4 in B2L (Fig. 5b,c).

Available strategies, such as homologue scanning or hybrid gene expression, can be applied to determine the general conformational epitope^21,27^. In the current study, binding assays using an individual phage on a purified target confirmed that the phage interacted with 2E4 specifically in a pattern consistent with its epitope. Therefore, random libraries might be advantageous when recombinant phages displaying infermation of epitopes are used for diagnosis or vaccination purposes^21^, as in our application. Using this approach for the mapping of epitope of a mAb, Wang and his assistants mapped an epitope on the bluetongue virus outer capsid protein VP5 in 1994–1995. In this paper, when the mimic epitope consensus motif VKVNPPQYDLE/RR was obtained, it was a fantastic and exciting challenge. Since very low sequence identity was found between the motif and the B2L gene, the 2E4 epitope was inferred to be a non-continued conformational pattern in the B2L protein. To identify the location of this conformational antigenic site, we expressed a set of truncated genes of B2L including F1, F2 and F3, and the interactivity of these products with 2E4 were assessed under the same reaction conditions. As expected, F1 displayed a unique capability for recognizing 2E4 through ELISA. None of these were detected on a nitrocellulose membrane by western blot, which corresponded to our expectations.

Epitope mapping is high consuming in terms of time and cost, making *in silico* methods a good complementary accelerant^28^. *In silico* studies and simulations can accelerate these research processes due to their time-saving manner^29^. High-accuracy predictions should result in a faster handling of the project being studied^30^*. In silico* approaches that showed the 3D-structure of B2L vastly facilitated our study of the 2E4 epitope. The 3D model of F1 showed 55 amino acid residues spatially in the front of B2L that contained 2α-helixes (Fig. 2b1), involved in five potential candidate peptides in the predicted epitope (Fig. 2a). Interestingly, the No.1:SSTKEGVDV- KDKLCTL (Score:0.85) and No.2:SKDKDADELRAAGINY (Score:0.83) that were generated via the *ABCpred* Prediction Server prediction appeared very close to ^84^VDVQSKDKDADELR^97^ of B2L. Perhaps, we merely need an eventual demonstration of these showing binding in a subsequent 2E4 binding test.

**Figure 6:**
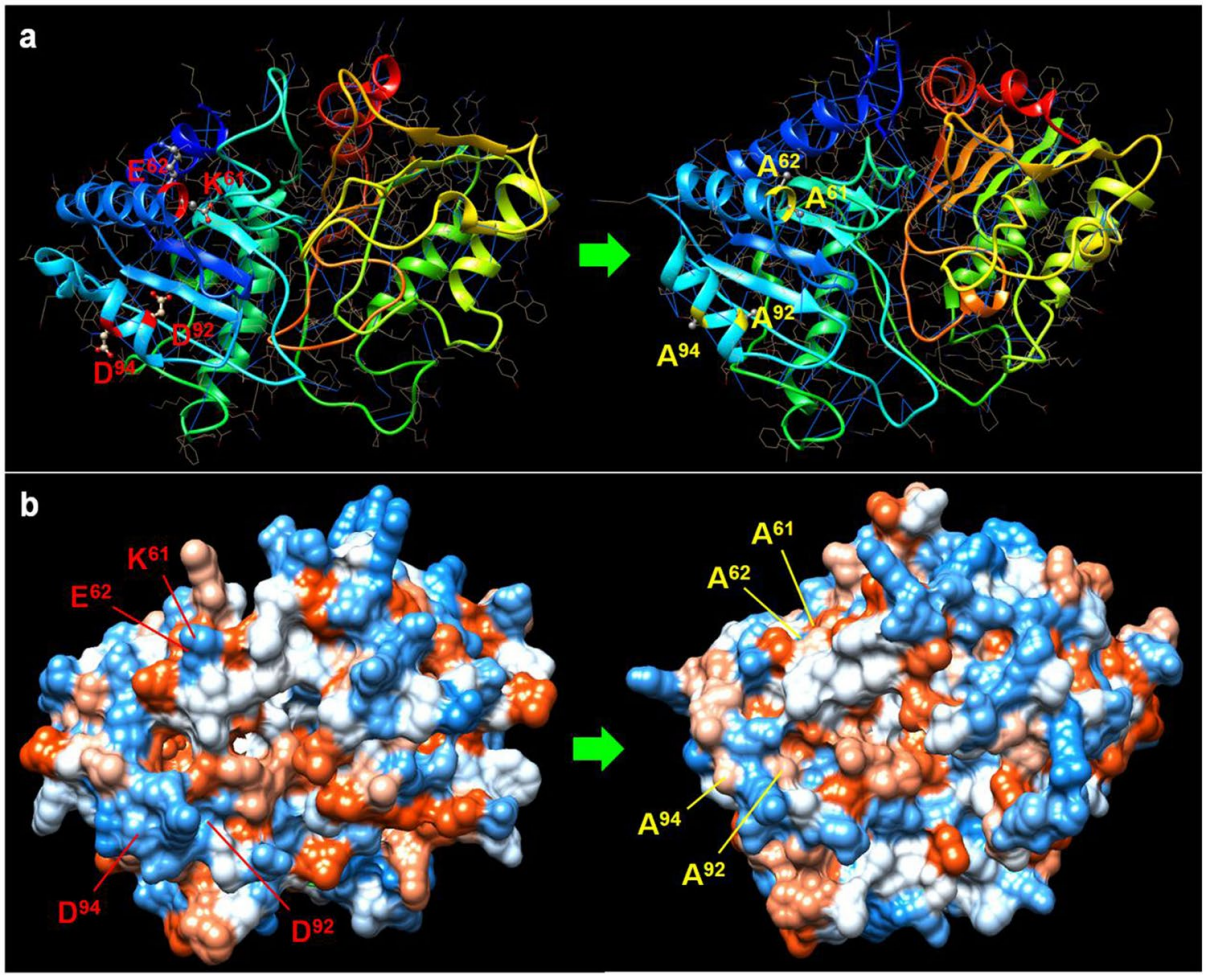
Amino acid substitution disrupts the structure of the 2E4 epitope. (**a**) The localization of K^61^, E^62^, D^92^ and D^94^ is displayed on structural modelling (on the left). After substitution of these four residues with alanines, binding to epitope was lost, and the B2L structure was reconstructed. The new model (on the right) is impacted compared with the original structure. (**b**) The 3D model displays hydrophobic properties. Due to the mutation, the new model (on the right) is slightly smaller than the original structure in size (on the left).

**Figure 7:**
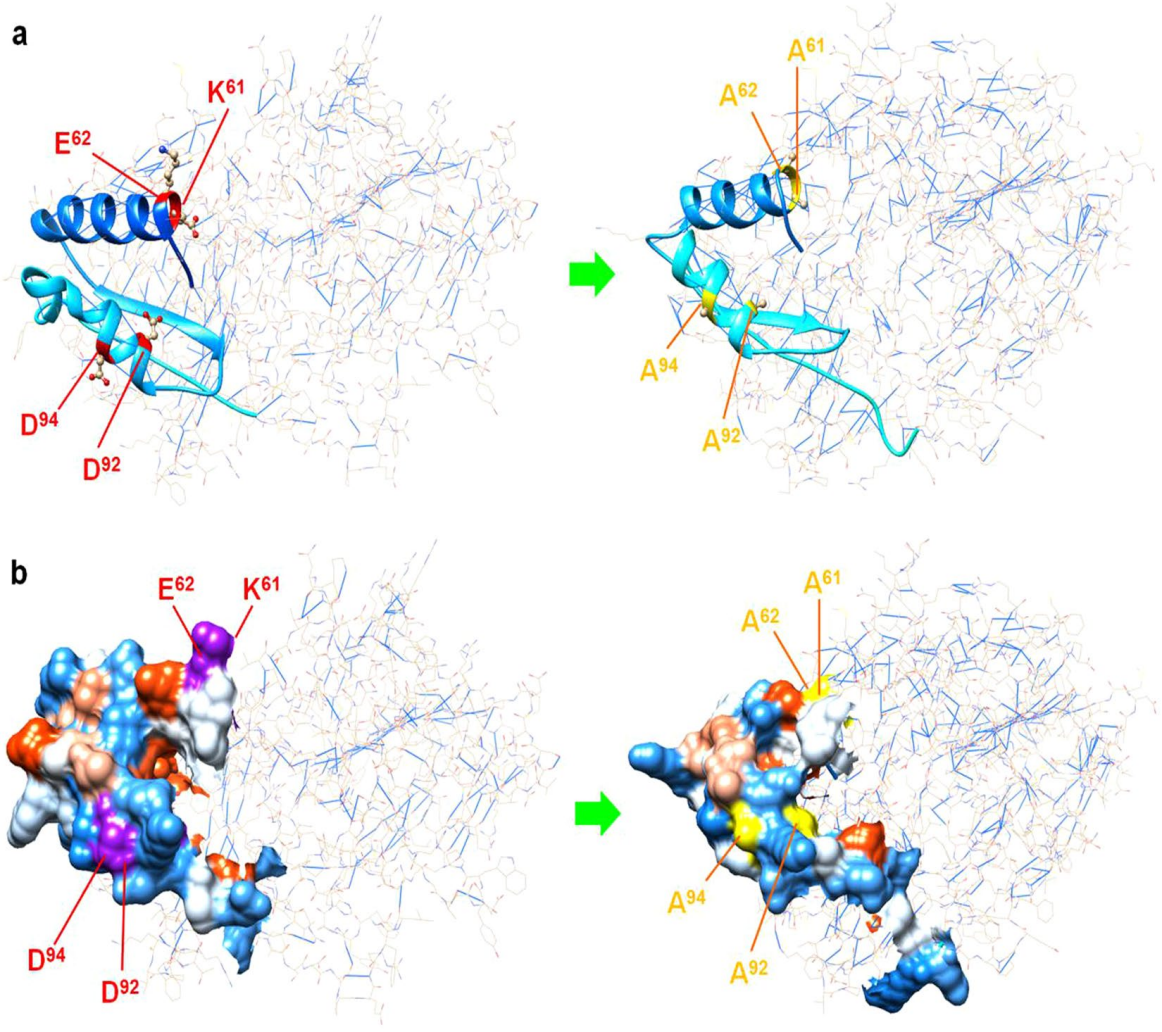
Structural mutant of the F1 domain due to amino acid substitution in homologous modelling. **(a)** To investigate the effectiveness of mutants in regard to their ability to affect the 2E4 epitope, clustered alanine substitution was used to test their contribution to the epitope. Mutation to alanine resulted in an obvious deformation of B2L involving the conformation of the F1 fragment. The hydrogen bond number between atoms was significantly increased in the new B2L model. **(b)** Deformation of B2L led to denaturation of the structure responsible for the binding activity to 2E4. Note: the red letters indicate replaced residues in the 2E4 epitope, while the yellow letters indicate nonvalent residues employed in this investigation.

**Figure 8:**
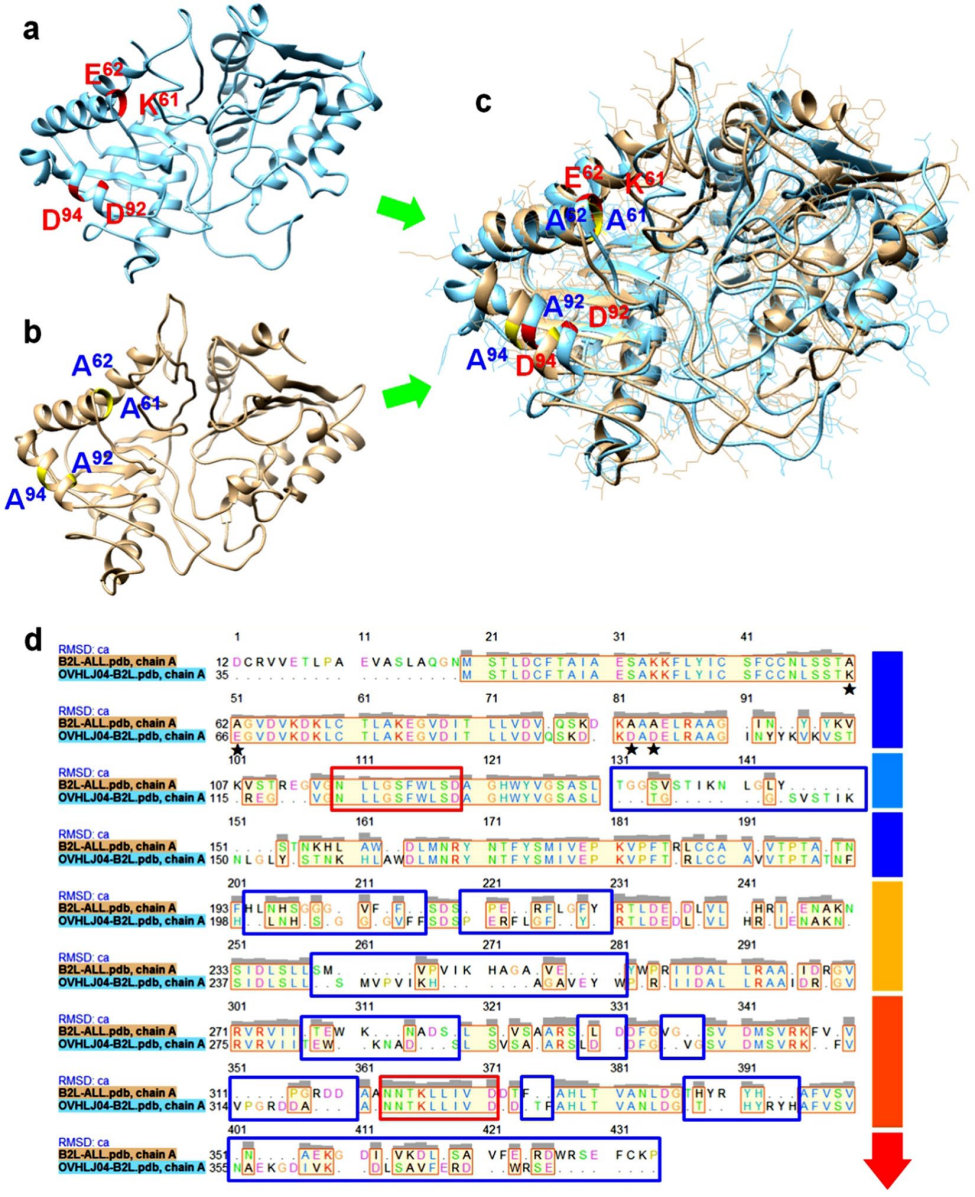
Comparison of the differences in the structural or functional domains based on the molecular conformation and the primary structure of the native B2L domain and the mutant. **(a)** The B2L molecule with the four major residues of the 2E4 epitope (K^61^, E^62^, D^92^ and D^94^). **(b)** The variant slightly influenced the molecular conformation of B2L via the four alanine residues. **(c)** Superimposed graph derived from the UCSF server suggesting that the essential structure of B2L persisted despite many additional loops emerging compared with the original structure. **(d)** Primary amino acid structure comparison between natural B2L and the mutant. We found that the mutant had not changed the folding pattern of the N-terminus that was occupied by the 2E4 epitope (the mutant residue is indicated by ⋆). However, the mutation resulted in extensive shifting of the locus in the latter part, particularly the C-terminus. Note: the two red rectangle frames indicate conserved functional regions; the multiple blue rectangle frames indicate the regions of the shifting locus. The coloured arrow indicates the variation tendency in structure stability.

Fortunately, among the three truncated peptides, F1 was observed to be specifically recognized by 2E4 by ELISA rather than by western blot (Figs 3e,f and s2). Experimental results have demonstrated that the mAb 2E4-recognized epitope is conformation-dependent, which may explain the low identity between the consensus motif VKVNPPQYDLE/RR and ^84^VDVQSKDKDADELR^97^ in the B2L protein (Fig. s1). Here, our 3D model highlighted that F1 is in the front of the B2L model, with all geographical possible residues of the epitope region (Fig. 5a–c). Around the tripeptide ^94^DEL^96^, we have found that D^92^ has structural significance, as well as that K^61^, K^69^ and K^75^ are related to its topological structure shown by alanine scanning mutagenesis (Fig. s3). Residue substitutions of K^61^, K^69^, K^75^, D^92^, E^95^and L^96^, had not directly affected the F1 binding activity to mAb 2E4 (Fig. 4d). Remarkably, the D^94^ residue mutant showed a distinctive detection value that impaired the binding activity of F1, and therefore was determined as a crucial residue for the ability of the epitope to bind to mAb 2E4. Furthermore, we wondered if K^61^, E^62^, D^92^ and D^94^ became an intact structure (namely “ALL”) responsible for 2E4 binding due to their very adjacent topological distribution (Fig. 4e). The ALL mutant nearly abolished the binding activity of F1 to mAb 2E4, which further showed this to be the most important element in the recognition of the epitope by mAb 2E4 (Figs 4e and 5c). Meanwhile, amino acid substitution confused the original structure of B2L, and the new model (on the right) is slightly smaller than the original structure (Fig. 6a,b). Additionally, the hydrogen bond number between atoms significantly increased in the new B2L model (Fig. 7a,b). However, the essential structure of B2L persisted, though many additional loops emerged compared with the original component (Fig. 8a–c). Mutagenesis of “ALL” resulted in extensive locus shifting in the B2L mutant, which was particularly serious in the C-terminus, and a structure-shifting trend was found (Fig. 8d).

From these results, we began to observe that the accuracy of the resulting 2E4 epitope constructs was verified by visualization servers (Fig. 5a–c). The prediction tools used here were computationally validated for identifying 2E4 epitope, contributing to the experimentally validated antigenic determinants. Many of the experimentally identified regions correctly overlapped with the computational predictions for the 2E4 epitope on F1. These findings emphasized the utility of *in silico* in mapping epitopes of mAbs.

## Materials and Methods

### Cells, virus and the mAb 2E4

MDBK cells were maintained in Dulbecco’s modified Eagles, medium (DMEM) supplemented with 10% heat-inactivated foetal bovine serum (FBS) (Gibco, Grand Island, NY) at 37 °C with 5% CO_2_. Hybridomas and mAbs to the B2L protein of ORFV were stored in our laboratory. The mAb 2E4 was preliminarily purified using the caprylic acid-ammonium sulfate method, followed by purification with the NAb^TM^ Protein G Spin Purification Kit (Pierce, USA).

### Biopanning

Selection of peptides from random peptide phage display libraries (Ph.D-12 Phage Display Peptide Library Kit, New England Biolabs) was described previously^22^. The affinity selection of the phage clones was conducted following the manufacturer’s recommendations with minor modifications. Briefly, in the first round, microwell plates were coated with 10 µg/ml of mAb 2E4 in 0.1 M NaHCO3 buffer (pH 8.6) at 4 °C for 12 h. The coated wells were washed with Tris-buffered saline (50 mM Tris-Cl pH 7.5, 150 mM NaCl) containing 0.1% Tween 20 (TBST) followed by blocking with 1 mg/ml bovine serum albumin (BSA) in 0.1 M NaHCO_3_ buffer. We added 2 × 10^10^ phages into the coated wells and incubated them with mAb-2E4 for 1 h at room temperature. Unbound phages were removed with 10 washes of TBST. The bound phages were eluted by 0.2 M glycine-HCl containing 1 mg/mL BSA (pH 2.2) and immediately neutralized with 1 M Tris-HCl (pH 9.1). The eluted phages were amplified in *E. coli* (ER2738) and titred on LB/IPTG/Xgal plates. The second and third rounds of selection were performed similarly to the first round, except the concentration of Tween-20 was raised to 0.5% while the concentrations of mAb 2E4 were reduced to 5 µg/mL, and 1 µg/mL, respectively. For the fourth round of biopanning, 300 ng of purified mAb 2E4 was mixed with the amplified third-round eluate (5 × 10^11^ phages) and incubated for 20 min at room temperature. Then, 50 mL of protein G-Agarose suspension (Gibco, NY, USA) was added, and the incubation was continued for 15 min at room temperature. The incubation mixture was centrifuged (30 s, 3,000 × g), and the supernatant was discarded while the pellet was washed 10 times with TBST. The final pellet was resuspended in 1 mL of 0.2 M glycine-HCl containing 1 mg/mL BSA (pH 2.2). The final eluted phages were plated, and individual clones were picked randomly for phage ELISA and DNA sequencing.

### *In vitro* phage binding assay

96-well plates were coated with 100 ng of purified mAb 2E4 (100 mL/well mAb in 0.1 M NaHCO_3_, pH 8.6) overnight at 4 °C. BSA solution was used as a negative control. The coated wells were blocked for 2 h at room temperature, and then the phages (10^10^ pfu/100 mL/well) diluted in blocking solution were added. The plates were incubated for 1 h at room temperature then washed 10 times with TBST. A phage clone was detected using horseradish peroxidase (HRP)-conjugated anti-M13 mAb (GE Healthcare, USA) at a 1:5000 dilution. The reaction was quantified using the ABTS [2,20-azinobis(3-ethylbenzthiaz -olinesulfonic acid)] substrate, and the absorbance at 450 nm was measured. Monophages exhibiting high absorbance were selected for sequencing. Single-stranded DNA was prepared according to the methods^24^ described previously and sequenced using the 96gIII sequencing primer.

### Sequencing and computer-based analyses of structure

After four additional rounds of selection of amplified phages, DNA from well-separated plaques was sequenced, and the corresponding peptide sequence was then generated from the DNA sequences. Computer analyses of DNA and protein sequences were performed using the *DNAStar Protean* software program (USA). The consensus sequence motif was defined by the peptide library screening. Molecular coordinates for the B2L protein used in structural analysis and modelling were obtained from the Protein Data Bank (PDB), then visualized and analysed using *Accelrys DS Visualizer 1.7*.

### Protokaryon expression plasmids and expression of antigenic peptides

For further epitope determination, we generated a series of antigenic fragments derived from B2L or B2L mutants by amino acid substitution to assess whether the reaction against the 2E4 mAb in a molecular context was different from that of the B2L protein. The three representative antigenic fragments F1 (aa at 58-112), F2 (aa at 205-235) and F3 (aa at 338-375) were expressed as recombinant fusions to GST, according to the antigenicity predication, B2L protein was used as a positive control, and a GST empty vector was used as a negative control, prepared using the Gateway® Cloning System (Thermo Fisher Scientific) according to manufacturer’s instructions. Briefly, the DNA sequences of interest were amplified using the primers containing the sequences shown in Table 1 (amino acid substitution) and Table 2 (fragments), followed by purification of the amplified products ready for gene expression according to manufacturer´s instructions (Thermo Fisher Scientific).

**Table 2:** Primer sequences of recombinant protein F1, F2 and F3 (restriction site is italic and underlined).

Name The Synthesized oligonucleotides	Restriction site
F1-F: 5-CG*GGATCC*AGCTCCACCAAGGAGGG-3	*BamH*I
F1-R: 5-CC*CTCGAG*TTCCCGCGTGGACACCT-3	*Xho*I
F2-F: 5-CG*GGATCC*TCGGACTCGCCGGA-3	*BamH*I
F2-R: 5-CC*CTCGAG*GTCGATGCTGTTCT-3	*Xho*I
F3-F: 5-CG*GGATCC*GACGGCACGCACTAC-3	*BamH*I
F3-R: 5-CC*CTCGAG*CTTGCAGAACTCCGA-3	*Xho*I

### Homology modelling and B-cell conformational epitope prediction

The amino acid sequences of B2L (GenBank accession number KU523790) were retrieved from the GenBank database and used as targets for homology modelling using the *SWISS MODEL* server (https://swissmodel.expasy.org/). The latter performed the target-template sequence alignment after searching the putative X-ray template proteins in PDB to generate the 3D models for all target sequences. The best homology models were selected according to Global Model Quality Estimation (GMQE) and QMEAN statistical parameters. Further, the established models were exported to the SAVES server Version 4 and their overall stereochemical quality, including backbone torsional angles through the Ramachandran plot, was checked according to PROCHECK. Lastly, each model was refined by an energy minimization protocol, using the Smart Minimizer algorithm in Discovery Studio version 4.1. Additionally, B2L-F1 was subjected to select probable epitopes using the *ABCpred* Prediction Server online (http://www.imtech.res.in/raghava/abcpred/index.html). The predicted B cell epitopes are ranked according to their score values obtained by a trained recurrent neural network. The higher peptide score means a higher likelihood of being an epitope. All peptides shown here are above the threshold value chosen.

### Western blot analyses on GST fusion proteins

For western blot analysis, approximately equal amounts of each GST fusion protein were subjected to 12% sodium dodecyl sulfate-polyacrylamide gel electrophoresis (12% SDS-PAGE). The gel was either stained with Coomassie blue staining solution or electrophoretically transferred to a nitrocellulose membrane. After blocking the membrane with 5% nonfat milk in PBS overnight at 4 °C, we incubated the membrane with mAb 2E4 (diluted 1:1,000 in PBS) at 37 °C for 1 h, then washed it three times with PBST, and probed it with a 1:2,000 dilution of HRP-conjugated goat anti-mouse IgG (Sigma) at 37 °C for 1 h. The reactivity was visualized with the substrate 3, 3′-diaminobenzidine (DAB; Sigma).

### Indirect ELISA

GST fusion protein was subjected to 2E4 for specificity and affinity detection. Plates were coated with 100 μl/well of purified GST fusion protein diluted in carbonate-bicarbonate buffer (pH 9.6) for incubation overnight at 4 °C, followed by four washes with 200 μl/well PBS/0.05% Tween-20 and blocking with 200 μl/well blocking buffer (PBS containing 5% skim milk) for 1 h at 37 °C. A total of 100 μl/well of the supernatant of the hybridoma was added in duplicate wells, and the plates were incubated for 1 h at 37 °C followed by the addition of horseradish peroxidase (HRP)-conjugated goat anti-mouse immuno-globulin G (IgG, Sigma) at a dilution of 1:2,000. After a 1 h incubation at 37 °C, 50 μl of substrate o-phenylene diamine dihydrochloride (OPD, Sigma) containing 0.3% H_2_O_2_ were added, and colour was developed for 15 min before stopping with 50 μl/well of 2 M H_2_SO_4_. Optical density was measured at 450 nm.

## Supplementary information

Dataset 1

Dataset 2

Dataset 3

Dataset 4

## References

[1] Al-Salam S, Nowotny N, Sohail MR, Kolodziejek J, Berger TG (2008). Ecthyma contagiosum (orf)–report of a human case from the United Arab Emirates and review of the literature. Journal of Cutaneous Pathology.

[2] Haig DM, Mercer AA (1998). Ovine diseases: Orf. Vet Res.

[3] Andrew A (2006). Comparative analysis of genome sequences of three isolates of Orf virus reveals unexpected sequence variation. Virus Research.

[4] Chan KW (2007). Identification and phylogenetic analysis of orf virus from goats in Taiwan. Virus Genes.

[5] Zhao K (2010). Identification and phylogenetic analysis of an Orf virus isolated from an outbreak in sheep in the Jilin province of China. Veterinary Microbiology.

[6] Sullivan JT, Mercer AA, Fleming SB, Robinson AJ (1994). Identification and characterization of an orf homologue of the vaccinia virus gene encoding the major envelope antigen p37K. Virology.

[7] Inoshima Y, Morooka A, Sentsui H (2000). Detection and diagnosis of parapoxvirus by the polymerase chain reaction. Journal of Virological Methods.

[8] Tsai SM (2009). Development of a loop-mediated isothermal amplification for rapid detection of orf virus. Journal of Virological Methods.

[9] Gallina L (2006). A real time PCR assay for the detection and quantification of orf virus. Journal of Virological Methods.

[10] Mortara L (1998). Selection of virus variants and emergence of virus escape mutants after immunization with an epitope vaccine. Journal of Virology.

[11] Hernandez H, Marceau C, Halliday H, Callison J, Rockx B (2015). Development and characterization of broadly cross-reactive monoclonal antibodies against all known Ebolavirus species. Journal of Infectious Diseases.

[12] Hovanessian AG (2004). The Caveolin-1 Binding Domain of HIV-1 Glycoprotein gp41 Is an Efficient B Cell Epitope Vaccine Candidate against Virus Infection. Immunity.

[13] Vider-Shalit T, Raffaeli S, Louzoun Y (2007). Virus-epitope vaccine design: Informatic matching the HLA-I polymorphism to the virus genome. Molecular Immunology.

[14] Depla E (2008). Rational design of a multiepitope vaccine encoding T-lymphocyte epitopes for treatment of chronic hepatitis B virus infections. Journal of Virology.

[15] Doehn C (2009). Mode-of-Action, Efficacy, and Safety of a Homologous Multi-Epitope Vaccine in a Murine Model for Adjuvant Treatment of Renal Cell Carcinoma. European Urology.

[16] Regenmortel MHVV (1996). Mapping Epitope Structure and Activity: From One-Dimensional Prediction to Four-Dimensional Description of Antigenic Specificity. Methods.

[17] Khudyakov YE (1993). Epitope Mapping in Proteins of Hepatitis E Virus. Virology.

[18] Zhang F (2006). Characterization of epitopes for neutralizing monoclonal antibodies to classical swine fever virus E2 and Erns using phage-displayed random peptide library. Archives of Virology.

[19] Yu YZ (2011). Fine mapping of a foot-and-mouth disease virus epitope recognized by serotype-independent monoclonal antibody 4B2. Journal of Microbiology.

[20] Zhang LM (2015). Identification of a Conserved Linear B-Cell Epitope of Streptococcus dysgalactiae GapC Protein by Screening Phage-Displayed Random Peptide Library. Plos One.

[21] Wang LF, Plessis DHD, White JR, Hyatt AD, Eaton BT (1995). Use of a gene-targeted phage display random epitope library to map an antigenic determinant on the bluetongue virus outer capsid protein VP5. Journal of Immunological Methods.

[22] Wang H, Zhao L, Li W, Zhou G, Yu L (2011). Identification of a conformational epitope on the VP1 G-H Loop of type Asia1 foot-and-mouth disease virus defined by a protective monoclonal antibody. Veterinary Microbiology.

[23] Irving MB, Pan O, Scott JK (2001). Random-peptide libraries and antigen fragment libraries for epitope mapping and the development of vaccines and diagnostics. Current Opinion in Chemical Biology.

[24] Kolaskar AS, Tongaonkar PC (1990). A semi-empirical method for prediction of antigenic determinants on protein antigens. Febs Letters.

[25] Mishra. A, Jain A, Arora N (2016). Mapping B-cell epitopes of major and minor peanut allergens and identifying residues contributing to IgE binding. J Sci Food Agric.

[26] Giordano RJ, Cardó-Vila M, Lahdenranta J, Pasqualini R, Arap W (2001). Biopanning and rapid analysis of selective interactive ligands. Nature Medicine.

[27] Rogers M, Serban D, Gyuris T, Scott M, Prusiner SB (1991). Epitope mapping of the Syrian hamster prion protein utilizing chimeric and mutant genes in a vaccinia virus expression system. Journal of Immunology.

[28] Kringelum JV, Lundegaard C, Lund O, Nielsen M (2012). Reliable B cell epitope predictions: impacts of method development and improved benchmarking. Plos Computational Biology.

[29] Poorinmohammad N, Mohabatkar H (2014). Homology Modeling and Conformational Epitope Prediction of Envelope Protein of Alkhumra Haemorrhagic Fever Virus. Journal of arthropod-borne diseases.

[30] Mohabatkar H, Keyhanfar M, Behbahani M (2012). Protein bioinformatics applied to virology. Current Protein & Peptide Science.

