# Supplementary material for: Structural Features of a Conformation-dependent Antigen Epitope on ORFV-B2L Recognized by the 2E4 mAb
Source: Sci Rep. 2019 Nov 6;9:16094. doi: 10.1038/s41598-019-52446-5 (PMC6834619; doi:10.1038/s41598-019-52446-5)
Supplement: Supplementary file 1 — Dataset 1 [file 41598_2019_52446_MOESM1_ESM.pdf]

Structural Features of a Conformation-dependent Antigen Epitope on ORFV-B2L Recognized by the 2E4 mAb

Yongzhong Yu[1]*, Wenbo Zhao[1], Qiang Tan[1], Xue Zhang[1], Mengyao Wang[1], Xuyang Duan[1], Yuanyuan Liu[1], Zhijun Wu[1], Jinzhu Ma[1], Baifen Song[1], Rui Zhao[2], Kui Zhao[3], Zhengxing Lian[4], Yudong Cui[1]*

[1] Virology Laboratory, College of Biological Science and Technology, Heilongjiang Bayi Agricultural University, 2 Xinyang road, Daqing 163319, China
[2] Pharmacology laboratory,Heilongjiang Bayi Agricultural University, 2 Xinyang road, Daqing 163319, China;
[3] College of Animal Science and Veterinary Medicine, Jilin University, 5333 Xi'an Road, Changchun 130062, China
[4] Beijing Key Laboratory for Animal Genetic Improvement, College of Animal Science and Technology, China Agricultural University, Beijing 100193, China

*Correspondence to yyz1968@126.com; cuiyudong@yahoo.com.

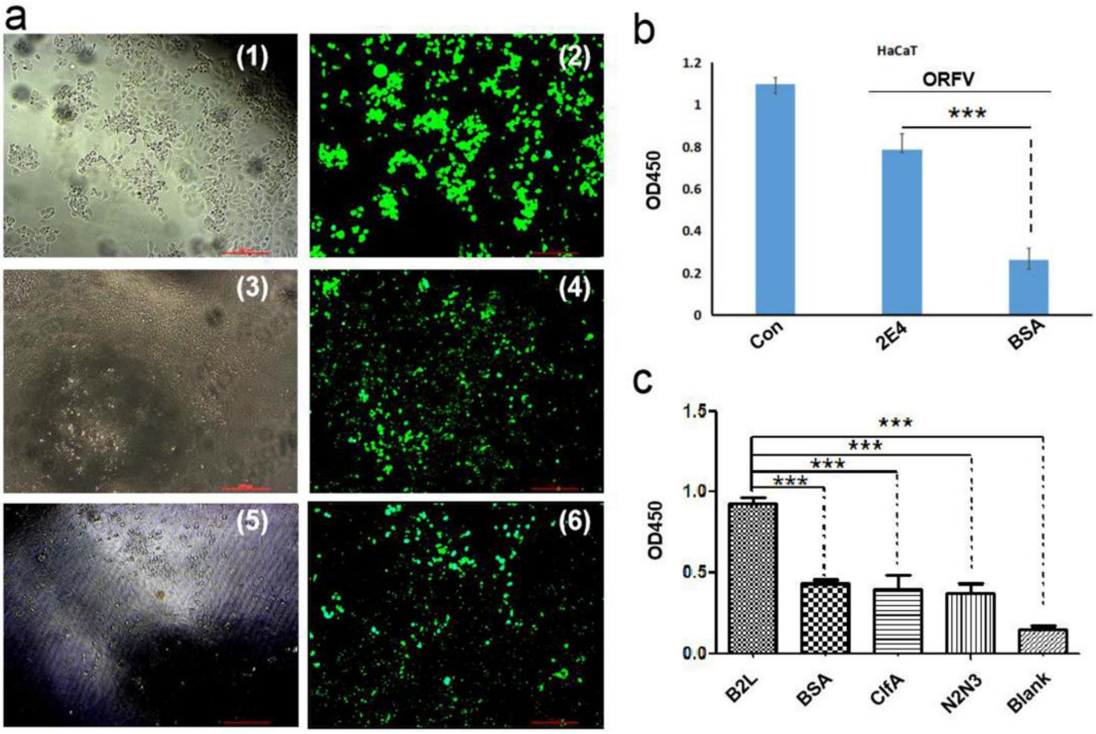

Figure s1. The characterisation of mAb 2E4 was determinated by functional assays. (**a**) Immunofluorescence assay (bar=200μm). (1) and (2) indicate HaCaT cells infected by ORFV 72h pi, and the 2E4 specially recognized ORFV within the infected cells. (3) and (4) indicate HaCaT cells infected by ORFV 72h pi, while 2E4 was administrated before the ORFV infection.(5) and (6) were controls without virus infection. (**b**) Neutralization test showed that 2E4 can protect cells from virus infection by ORFV rather than BSA and in (**c**), 2E4 only recognized B2L protein among with a significant specificity to ORFV. (∗∗∗: $p < 0.001$)
