# Supplementary material for: Structural Features of a Conformation-dependent Antigen Epitope on ORFV-B2L Recognized by the 2E4 mAb
Source: Sci Rep. 2019 Nov 6;9:16094. doi: 10.1038/s41598-019-52446-5 (PMC6834619; doi:10.1038/s41598-019-52446-5)
Supplement: Supplementary file 2 — Dataset 2 [file 41598_2019_52446_MOESM2_ESM.pdf]

Structural Features of a Conformation-dependent Antigen Epitope on ORFV-B2L Recognized by the 2E4 mAb

Yongzhong Yu[1]*, Wenbo Zhao[1], Qiang Tan[1], Xue Zhang[1], Mengyao Wang[1], Xuyang Duan[1], Yuanyuan Liu[1], Zhijun Wu[1], Jinzhu Ma[1], Baifen Song[1], Rui Zhao[2], Kui Zhao[3], Zhengxing Lian[4], Yudong Cui[1]*

[1] Virology Laboratory, College of Biological Science and Technology, Heilongjiang Bayi Agricultural University, 2 Xinyang road, Daqing 163319, China
[2] Pharmacology laboratory,Heilongjiang Bayi Agricultural University, 2 Xinyang road, Daqing 163319, China;
[3] College of Animal Science and Veterinary Medicine, Jilin University, 5333 Xi'an Road, Changchun 130062, China
[4] Beijing Key Laboratory for Animal Genetic Improvement, College of Animal Science and Technology, China Agricultural University, Beijing 100193, China

*Correspondence to yyz1968@126.com; cuiyudong@yahoo.com.

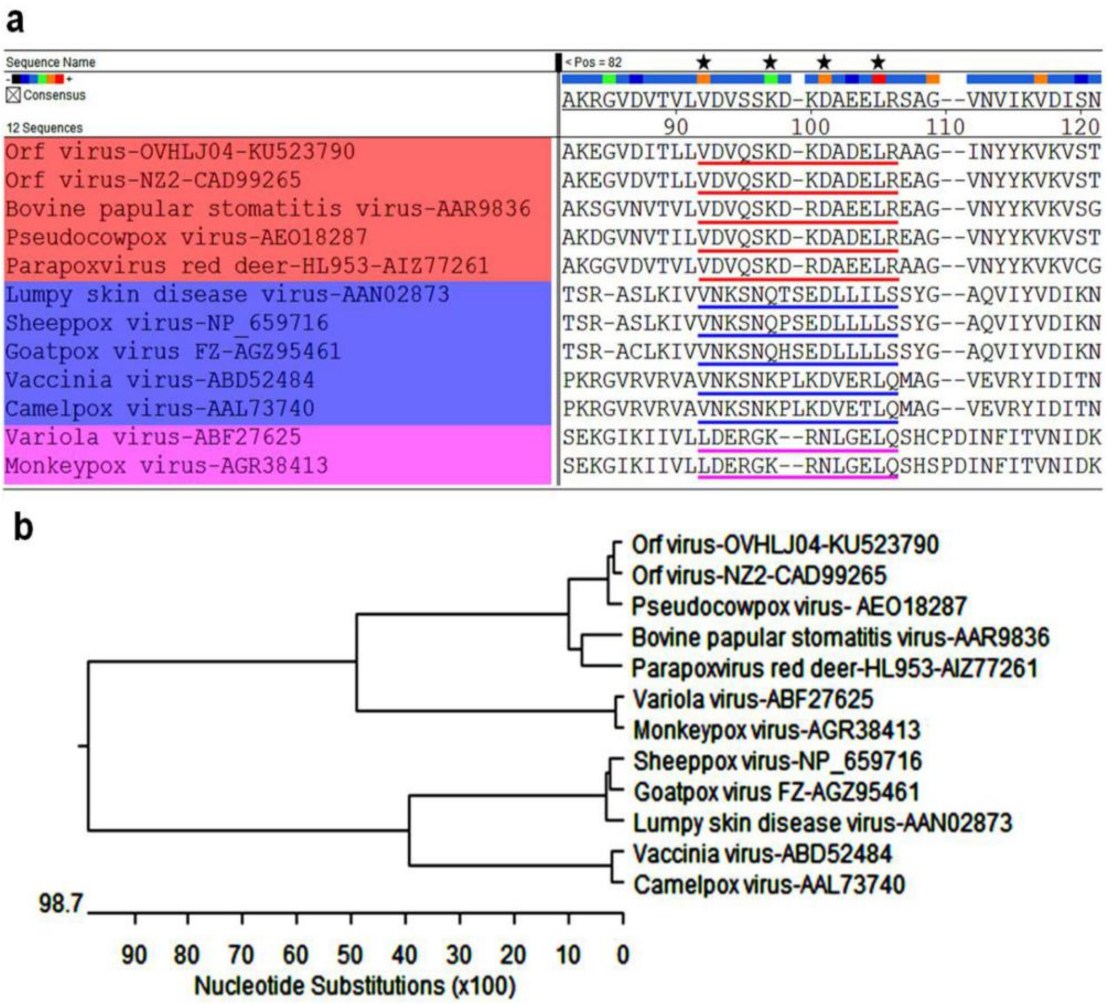

Figure s2. Partial sequence analysis and phylogenetic tree of B2L gene of ORFV with the representative members of family *Poxvirus*.(**a**) Multiple alignments. A total of 12 sequences of *Poxvirus* derived from NCBI with their accession numbers were simultaneously aligned using DNAStar-MegAlign software. They were divided into three regions by different colors and, the pale red, baby blue and light pink represented various genetic relationships within the family, respectively. The tetradecapeptide VDVQSKDK(R)DADELR of genus *Parapoxvirus* was attractile with its conservative property in the red region, and holded a lower identity compared with other regions. In addition, relative conservative amino acids were indicated by "black five-pointed stars" on the top of image. (**b**) Phylogenetic analysis were conducted using DNAStar. The *Poxvirus* isolates (from GenBank) that exhibited similarity to ORFV/OVHLJ04 (KU523790), were subjected to evaluate the phylogenetic relationship for multiple sequence alignments.
