# Supplementary material for: Structural Features of a Conformation-dependent Antigen Epitope on ORFV-B2L Recognized by the 2E4 mAb
Source: Sci Rep. 2019 Nov 6;9:16094. doi: 10.1038/s41598-019-52446-5 (PMC6834619; doi:10.1038/s41598-019-52446-5)
Supplement: Supplementary file 3 — Dataset 3 [file 41598_2019_52446_MOESM3_ESM.pdf]

Structural Features of a Conformation-dependent Antigen Epitope on ORFV-B2L Recognized by the 2E4 mAb

Yongzhong Yu[1]*, Wenbo Zhao[1], Qiang Tan[1], Xue Zhang[1], Mengyao Wang[1], Xuyang Duan[1], Yuanyuan Liu[1], Zhijun Wu[1], Jinzhu Ma[1], Baifen Song[1], Rui Zhao[2], Kui Zhao[3], Zhengxing Lian[4], Yudong Cui[1]*

[1] Virology Laboratory, College of Biological Science and Technology, Heilongjiang Bayi Agricultural University, 2 Xinyang road, Daqing 163319, China
[2] Pharmacology laboratory,Heilongjiang Bayi Agricultural University, 2 Xinyang road, Daqing 163319, China;
[3] College of Animal Science and Veterinary Medicine, Jilin University, 5333 Xi'an Road, Changchun 130062, China
[4] Beijing Key Laboratory for Animal Genetic Improvement, College of Animal Science and Technology, China Agricultural University, Beijing 100193, China

*Correspondence to yyz1968@126.com; cuiyudong@yahoo.com.

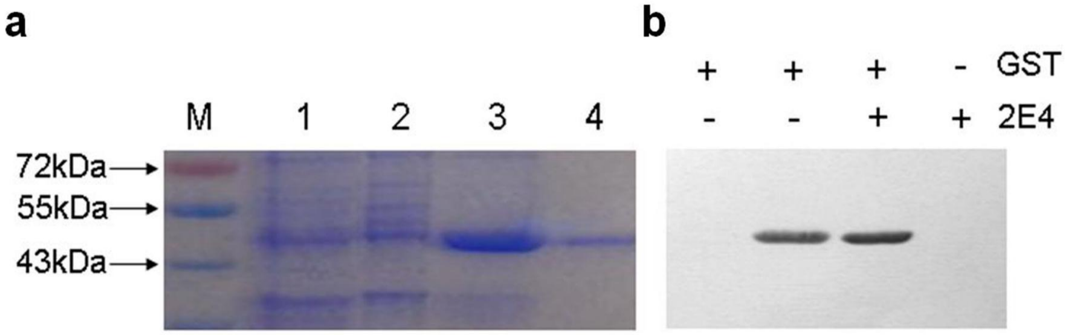

Figure s3. SDS-PAGE and western blot analysis to determine the B2L expression and binding activity to mAb 2E4. **(a)** The GST-fused proteins were separated by 12% SDS-PAGE, and the gel was stained with Coomassie Blue. M: Molecular weight marker; 1: Whole-cell lysates of *E. coli* BL21 (DE3); 2,3: Expressed products of recombinant plasmids were induced for 1 to 3h with isopropyl β -D-1-thiogalacto-pyranoside (IPTG); 4: Purified B2L protein samples. **(b)** Western blot analysis corresponding to the fusion proteins shown in (a). lane 1: The lysates showed no reaction to anti-GST Tag mAb (Thermo fisher); lane 2,3: The prducts of post-induction were all recognized by GST Tag antibody. Nevertheless, lane 4: The purified B2L was failing to recognize 2E4 by western blot.
