# Supplementary material for: Structural Features of a Conformation-dependent Antigen Epitope on ORFV-B2L Recognized by the 2E4 mAb
Source: Sci Rep. 2019 Nov 6;9:16094. doi: 10.1038/s41598-019-52446-5 (PMC6834619; doi:10.1038/s41598-019-52446-5)
Supplement: Supplementary file 4 — Dataset 4 [file 41598_2019_52446_MOESM4_ESM.pdf]

Structural Features of a Conformation-dependent Antigen Epitope on ORFV-B2L Recognized by the 2E4 mAb

Yongzhong Yu[1]*, Wenbo Zhao[1], Qiang Tan[1], Xue Zhang[1], Mengyao Wang[1], Xuyang Duan[1], Yuanyuan Liu[1], Zhijun Wu[1], Jinzhu Ma[1], Baifen Song[1], Rui Zhao[2], Kui Zhao[3], Zhengxing Lian[4], Yudong Cui[1]*

[1] Virology Laboratory, College of Biological Science and Technology, Heilongjiang Bayi Agricultural University, 2 Xinyang road, Daqing 163319, China
[2] Pharmacology laboratory,Heilongjiang Bayi Agricultural University, 2 Xinyang road, Daqing 163319, China;
[3] College of Animal Science and Veterinary Medicine, Jilin University, 5333 Xi'an Road, Changchun 130062, China
[4] Beijing Key Laboratory for Animal Genetic Improvement, College of Animal Science and Technology, China Agricultural University, Beijing 100193, China

*Correspondence to yyz1968@126.com; cuiyudong@yahoo.com.

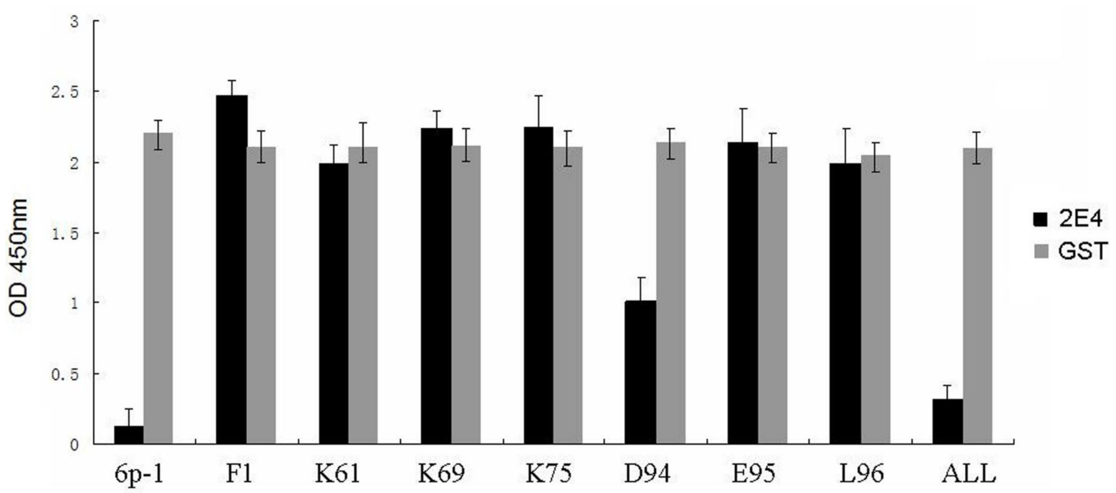

Figure s4.Various mutants with their detection values in ELISA. A set of ELISAs were achieved for 2E4 epitope and compared with expression vectors to anti-GST Tag mAb, respectively. GST Tag protein was used as a control experimentally. “All” represents variant of which K^{61}, E^{62}, D^{92} and D^{94} were substituted by four alanines simultaneously.
